# A practical guide to botulinum neurotoxin treatment of shoulder spasticity 1: Anatomy, physiology, and goal setting

**DOI:** 10.3389/fneur.2022.1004629

**Published:** 2022-10-17

**Authors:** Jorge Jacinto, Alexandre Camões-Barbosa, Stefano Carda, Damon Hoad, Jörg Wissel

**Affiliations:** ^1^Centro de Medicina de Reabilitação de Alcoitão, Serviço de Reabilitação de Adultos 3, Alcabideche, Portugal; ^2^Centro Hospitalar Universitário de Lisboa Central, Lisbon, Portugal; ^3^Centre Hospitalier Universitaire Vaudois (CHUV), Neuropsychology and Neurorehabilitation, Lausanne, Switzerland; ^4^Warwick Medical School, University of Warwick, Coventry, United Kingdom; ^5^Department of Neurorehabilitation and Physical Therapy, Vivantes Hospital Spandau, Berlin, Germany

**Keywords:** botulinum (neuro)toxin, muscle spasticity, shoulder, injection, pain

## Abstract

Botulinum neurotoxin type A (BoNT-A) is a first-line treatment option for post-stroke spasticity, reducing pain and involuntary movements and helping to restore function. BoNT-A is frequently injected into the arm, wrist, hand and/or finger muscles, but less often into the shoulder muscles, despite clinical trials demonstrating improvements in pain and function after shoulder BoNT-A injection. In part 1 of this two-part practical guide, we present an experts' consensus on the use of BoNT-A injections in the multi-pattern treatment of shoulder spasticity to increase awareness of shoulder muscle injection with BoNT-A, alongside the more commonly injected upper limb muscles. Expert consensus was obtained from five European experts with a cumulative experience of more than 100 years of BoNT-A use in post-stroke spasticity. A patient-centered approach was proposed by the expert consensus: to identify which activities are limited by the spastic shoulder and consider treating the muscles that are involved in hindering those activities. Two patterns of shoulder spasticity were identified: for Pattern A (adduction, elevation, flexion and internal rotation of the shoulder), the expert panel recommended injecting the pectoralis major, teres major and subscapularis muscles; in most cases injecting only the pectoralis major and the teres major is sufficient for the first injection cycle; for Pattern B (abduction or adduction, extension and internal rotation of the shoulder), the panel recommended injecting the posterior part of the deltoid, the teres major and the latissimus dorsi in most cases. It is important to consider the local guidelines and product labels, as well as discussions within the multidisciplinary, multiprofessional team when deciding to inject shoulder muscles with BoNT-A. The choice of shoulder muscles for BoNT-A injection can be based on spastic pattern, but ideally should also firstly consider the functional limitation and patient expectations in order to establish better patient-centered treatment goals. These recommendations will be of benefit for clinicians who may not be experienced in evaluating and treating spastic shoulders.

## Introduction

Spasticity is a common occurrence after stroke, traumatic brain injury, spinal cord injury, or multiple sclerosis. Spastic movement disorder, as defined by Pandyan et al. (1) and herein termed “spasticity”, consists of all positive symptoms of upper motor neuron syndrome (UMNS) – normally characterized by increased levels of involuntary motor activity, and including spasticity (a velocity-dependent increase in muscle tone) and spastic dystonia (1–3). Upper limb (UL) spasticity has been reported to affect as many as 43% of patients up to 12 months post-stroke (4, 5) and may be associated with pain following stretching of the muscles and soft tissue involved, deformity, impairment and/or loss of limb function (6, 7). This can negatively impact quality of life (QoL) due to the need to depend on others (8) as well as resulting in social isolation and depression (9–11).

Botulinum neurotoxin A (BoNT-A) is first-line treatment for focal, multifocal and segmental spasticity and provides pain relief for those with spasticity-associated pain, reduces involuntary movements in UMNS and helps to restore both passive and active function (7, 12, 13). Furthermore, treatment with BoNT-A has been shown to improve patient QoL and has a superior cost-utility ratio compared with conventional oral antispastic therapy in UL spasticity (14). Most pivotal studies of BoNT-A to treat UL spasticity focused on injecting arm, wrist, hand and/or finger muscles and did not include shoulder muscles (15–20), with only two UL spasticity trials including the shoulder (21, 22). Furthermore, shoulder muscles are seldom injected with BoNT A in the real-world management of UL spasticity (23) despite hemiplegic shoulder pain being the most common post-stroke pain disorder (24) and evidence supporting the possible reduction of this pain by shoulder muscle injection with BoNT-A (7, 21, 22, 25–27). Shoulder spasticity can restrict the functional capacity of the UL, especially the ability of patients to reach for objects such as a light switch, or a doorknob. Problems with axillary hygiene and dressing, as well as body image can impact patients' self-esteem. In turn, this may impact their confidence or independence to socialize, and limits how they can explore their environment, express themselves and participate in their community.

Shoulder muscles have been included in some clinical trials of BoNT-A in UL spasticity (11, 21, 22, 25, 26, 28) and improvements in shoulder spasticity were observed after treatment (11). Injection with BoNT-A was also shown to improve shoulder function and reduce pain in an observational study of chronic post-stroke spasticity (29). Despite this evidence, injecting shoulder muscles represents a major change in the common approach to the management of UL spasticity with BoNT-A and there has been some concern over the complexity of accurate injections, depending on the technique used, particularly in deeper muscles of the limb and trunk (30). Given the benefits of this treatment approach, it is important to increase the awareness of why shoulder muscle injection with BoNT-A should be considered by clinicians, alongside the more commonly injected UL muscles. This is particularly important for those who may not be familiar or confident enough with treating shoulder muscles, and training is needed on approaches to goal setting, choice of goal-related muscles and injection guidance techniques. This article provides the reader with clinical practice points to address this issue. Collective experience of more than 100 years of BoNT-A use by expert injectors is synthesized and we provide recommendations to overcome the challenges of multi-pattern shoulder BoNT-A injections for shoulder spasticity in this and an accompanying manuscript.

This first manuscript focuses on structural and functional anatomy, motor control and synergies in UMNS, as well as goal setting. In the accompanying manuscript we address BoNT-A injection technique and choice of outcome measurement scales, as well as providing instructive case studies to demonstrate how the treatment of shoulder spasticity and the injection guidance can be applied in practice.

## Methods

A two-part expert meeting was held online via video conference calls in October and November 2021 (3 h per video conference call). Five European experts who are members of university and teaching hospitals and national as well as international medical advisory boards in physical medicine and rehabilitation and neurological rehabilitation associations with a cumulative experience of more than 100 years in post-stroke spasticity gave focused presentations on shoulder spasticity and treatment with BoNT-A injections followed by discussion.

Topics included structural and functional anatomy, synergies, goal setting, injection techniques and case studies. In order to maximize the productivity of the meeting, pre-meeting surveys were conducted to capture information on each expert's preferred treatment practices. The surveys were drafted on behalf of the sponsor and included open-ended questions to drive the discussion sections of the meeting. The expert panel were asked to identify which patterns of UL spasticity were most commonly encountered in their daily practice, which shoulder muscles they treat for each pattern and which activities are impaired by the most common and rare shoulder spasticity patterns following stroke. The panel were also asked for their top short- and long-term goals for treatment of UL spasticity with shoulder involvement and recommended clinical evaluation scales.

The discussion was conducted in a way to identify consensus between treatment practices and to provide treatment recommendations based on this consensus. The online meetings were held with the intention of producing two linked manuscripts to present the findings as a practical guide for treating shoulder spasticity for the international neurorehabilitation community.

### Structural and functional anatomy

The main role of the shoulder is to move the UL so that the distal part is sufficiently positioned to bring the hand into the optimal workspace as needed (31). Consequently, disruption to shoulder function has an impact on accurate UL movement, which in most cases limits hand function. An impaired shoulder also affects emotional expressions and social behavior (e.g., an embrace).

**Figure 1:**
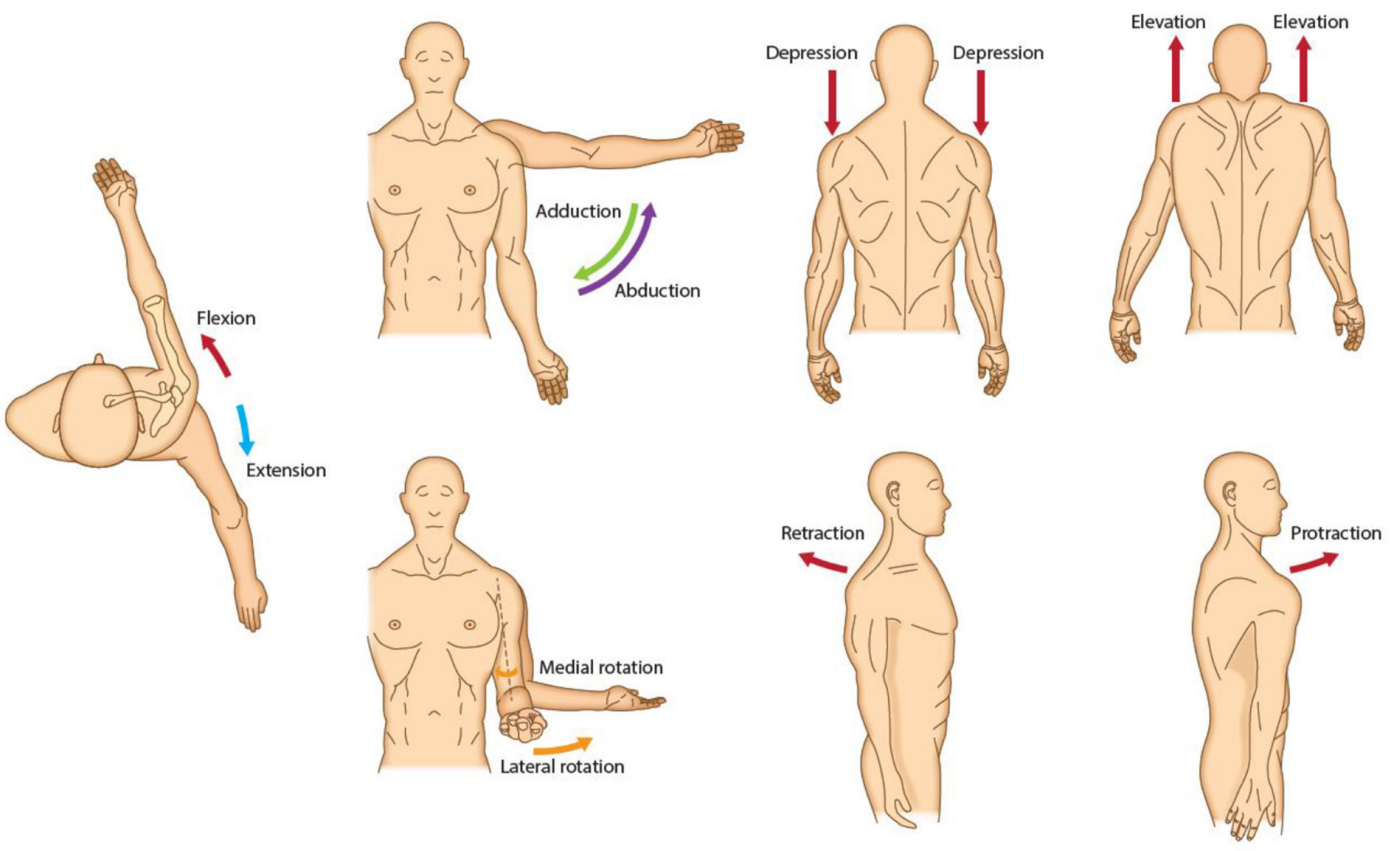
Upper limb degrees of freedom at shoulder.

**Figure 2:**
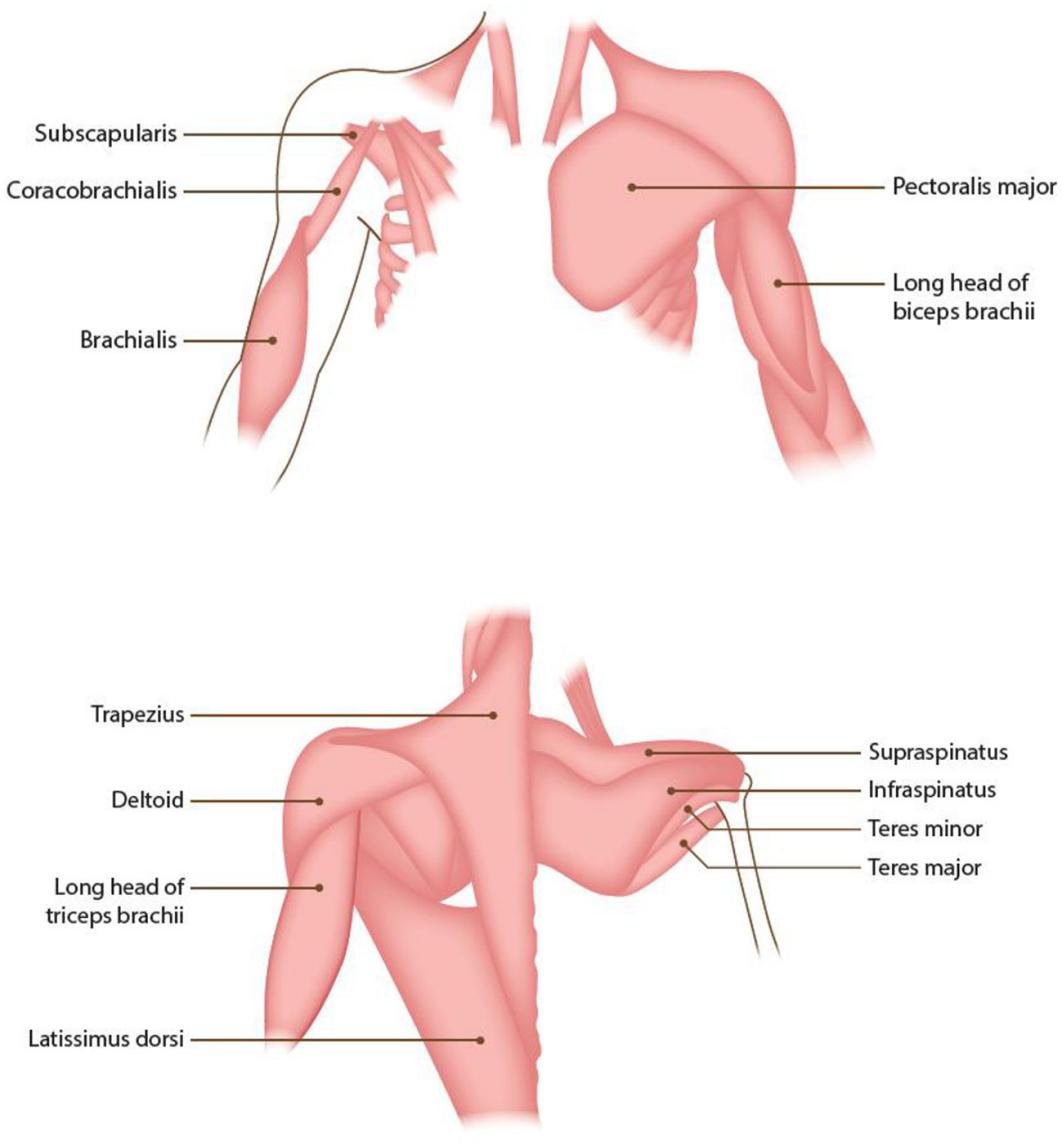
Anatomy of the shoulder muscles.

The shoulder moves multi-axially through combinations of elevation/depression, protraction/retraction, flexion/extension, medial/lateral rotation and abduction/adduction (Figure 1). The shoulder muscles include the deltoid, trapezius, teres major and minor, pectoralis major, subscapularis, supra- and infra-spinatus, coracobrachialis, latissimus dorsi and the long head of the biceps and triceps brachii muscles (Figure 2) (31). Five typical patterns of post-stroke UL spasticity that, cover more than 95% of disfigurement encountered in clinical practice when managing stroke survivors, have been previously described by an expert consensus group (Patterns I–V). Regarding shoulder involvement, four of the five patterns are a result of spastic flexion, internal rotation and adduction of the shoulder, whereas the fifth pattern is a result of abduction or adduction, internal rotation and extension (32).

**Figure 3:**
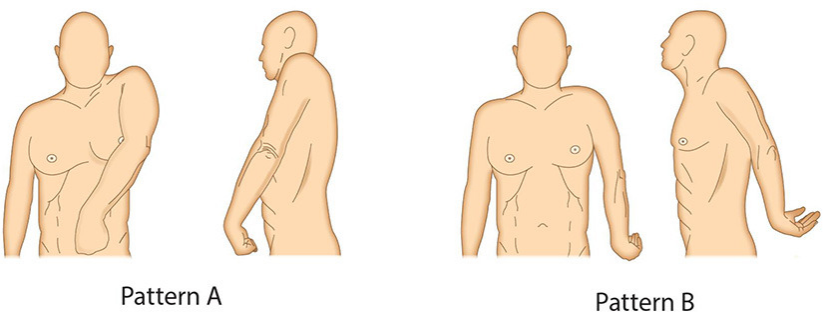
Two most common shoulder spasticity patterns. **(A)** Adduction, elevation, flexion and internal rotation of shoulder. **(B)** Abduction or adduction, extension and internal rotation of shoulder.

Post-stroke, Pattern III was most commonly encountered in the experts' daily practice, in agreement with previous publications (32, 33), followed by Pattern I. The least common pattern was Pattern V; that is, internal rotation and extension of the shoulder, again in agreement with previous data (32). The expert group adapted the five UL spasticity patterns of Hefter et al. (32) into two shoulder spasticity patterns: “A” (adduction, elevation, flexion and internal rotation of shoulder) and “B” (abduction or adduction, extension and internal rotation of shoulder; Figure 3); the difference between the two patterns is the presence of elevation and flexion in Pattern A vs. extension in Pattern B.

The traditional approach to choosing which shoulder muscle(s) to inject is to focus on the dominant presented spastic pattern and then dedicate attention to the underlying functional anatomy. For Pattern A, the expert panel recommended injecting the pectoralis major and teres major in most cases for the first injection cycle. Second-line recommendations included the subscapularis and latissimus dorsi. The pectoralis major is the major ventral shoulder adductor and together with the teres major – the major dorsal shoulder adductor – these muscles represent the major shoulder adductors. The teres major and the latissimus dorsi are also strong internal rotators and are easier to inject than the subscapularis, the strongest internal rotator (34, 35). For Pattern B, the expert panel most frequently recommended injecting the posterior part of the deltoid if abduction is present, and the teres major and the latissimus dorsi if adduction is present; the subscapularis can also be involved (Table 1). In patients with active function or capacity to use shoulder movements in daily life, we propose a novel, more practical and patient-centered approach, which begins by identifying which activities are impaired by the spastic shoulder pattern and considers treating the muscles that are involved in hindering those activities. Both approaches can be used in combination with each other. For example, the spastic pattern can identify which muscles are affected, and then the patient-centric approach can be used to identify which of these muscles should be treated in order to best achieve the patient's goals. This approach is also recommended for passive function (which drives the most common goals in this patient population) as well as for symptomatic and postural goals. It is important to be aware that peripheral factors can have an impact on the perceived spasticity pattern, particularly in patients with chronic spasticity. For example, patients may be using slings to prevent capsular distension and reduce pain, which over time can result in deltoid muscle atrophy and affect posture independently of the spastic muscles. Furthermore, UL position can vary for rest/sitting vs. walking, potentially altering the observed spasticity pattern (36). Table 2 shows which muscles are typically affected for commonly reported impaired activities. This is a general indication, but the analysis of which muscles to inject should always be done according to the individual patient's needs.

**Table 1:** Relationship of shoulder muscles with upper limb spasticity patterns for identifying candidate shoulder muscles for BoNT-A injection.

**Recommendation**	**Shoulder spasticity pattern**	
	**A: Adduction, elevation, flexion, and internal rotation of shoulder**	**B: Abduction or adduction, extension, and internal rotation of shoulder**
**Inject in most cases/**	Pectoralis major	Posterior part of deltoid^a^
**first injection cycle**	Teres major	Teres major^b^
		Latissimus dorsi^b^
**Inject in some**	Subscapularis	Subscapularis
**cases/second-line injection**	Latissimus dorsi	Long head of triceps brachii
	Long head of biceps brachii	

a Inject in most cases for patients with abduction and extension (combined with injection to the intermediate deltoid), but not in patients with internal rotation; inject in some cases for patients with adduction, extension and internal rotation.

b Inject if adduction, extension and internal rotation are present.

BoNT-A, botulinum neurotoxin type A.

**Table 2:** Relationship of shoulder muscles with impaired activities/symptoms for identifying candidate shoulder muscles for BoNT-A injection.

**Muscle**	**Prevalent pain in shoulder**	**Dressing (upper limb)**	**Reaching**	**Hygiene/grooming**	**Destabilized gait**
**Deltoid**			✓		✓
**Pectoralis major**	✓	✓	✓	✓	✓
**Subscapularis**	✓	✓	✓	✓	
**Latissimus dorsi**	✓	✓	✓	✓	✓
**Teres major**	✓	✓	✓	✓	
**Biceps brachii**		✓	✓		✓

BoNT-A, botulinum neurotoxin type A.

In general, when the major spastic muscles are treated, greater control of shoulder movement can be attained, and passive and active function, as well as symptom control and involuntary movements, will improve. Local guidelines must be considered, which may also guide or limit how many muscles can be injected with BoNT-A plus how frequently and at what dose BoNT-A can be administered, in addition to following the respective BoNT-A label for your region.

Finally, the decision to inject the shoulder muscles with BoNT-A should ideally be taken within the multidisciplinary team comprising healthcare professionals from across different specialities. Other strategies and interventions need to be considered, such as physiotherapy, occupational therapy, splinting, casting and physical agents (e.g., heat, cold, vibration, electrical stimulation, shock waves). Adequate state of the art management of post-stroke spasticity requires an integrated comprehensive multimodal approach and a multidisciplinary team, who must work together to optimize the patient's goal achievement and perception of benefit–outcome.

### Motor control and synergies

Motor control relies on all levels of the central nervous system, with representation of movements at cortical, subcortical, brainstem and spinal levels (37). Synergies are basic patterns of neural activation and inactivation of groups of muscles involved in common motor tasks, providing a computationally efficient way of coordinating axial, proximal and distal muscles, prime movers and stabilizers, agonists and antagonists. A simple motor command can control muscle patterning across many spinal levels. Combinations of just a few synergies are needed to control reach and gait. However, when neural motor pathways are damaged, this ability to activate or inactivate patterns of multiple muscles can result in spastic and dystonic postures. Because proximal stability is essential to allow the hand to function in a useful workspace, patterning shoulder muscle activation is a vital function of synergy control (38).

Synergies are controlled in movement representations on the cortical, subcortical, brainstem and spinal levels. Stroke can result in synergy controls becoming merged or fractionated. Common patterns of abnormal synergies are frequently observed, such as flexor synergies, where attempted elbow flexion drives shoulder abduction.

The location and dimensions of the stroke lesion will determine the impact on synergy control. Lesion site will influence both the extent of cortical reorganization of synergy representations, and the subsequent balance of pyramidal and extrapyramidal descending motor drive to spinal level synergy control. Multi-pattern injection of BoNT-A to spastic shoulder muscles offers the chance to rebalance abnormal synergy activation. Recognizing the involved synergies, translating those to muscles and treating the affected muscles is key to patient benefit.

Over time after stroke, biological recovery of pyramidal pathways may restore balance between ipsilateral and contralateral motor drive to the spinal circuits distributing synergy commands (the propriospinal system). Sensory feedback is an important input to this synergy-driving spinal network system, and this can be influenced by BoNT-A treatment.

Additional to synergy representation of motor control is the myotatic unit, represented at spinal levels. Sensory input can activate all muscles with tendon insertions acting on a joint (39). When motor control is disrupted, a sensory stimulus may coactivate multiple muscles in a myotatic unit to act in unison. For commonly encountered patterns of shoulder spasticity, such as internal rotation and adduction, targeting all inappropriately activated muscles in that unit is required to break the spastic pattern. Reducing this overactivity in exaggerated myotatic units can then alter the sensory afference, change input to propriospinal systems and help to reorganize functional shoulder movement to a better functional level. Thus, after beginning with the suggested standard treatment approach, treatment optimization can be achieved by clinical follow-up on synergies, reorganization of motor control and influence of sensory input, resulting in flexible adaptation of multipattern BoNT-A therapy over time.

### Goal setting

Goal setting is an essential part of the multidisciplinary therapeutic process when treating patients with spasticity. Goals should be patient-centric and the patient and their family/caregiver should therefore be involved in deciding what they want to achieve from treatment and in what time frame (12). Goal setting provides psychological engagement and benefit in terms of hope and motivation for the patient, which in turn empowers patients in their recovery programme (40). The Goal Attainment Scale can be used for goal setting and assessment, and is discussed further in the accompanying manuscript.

Impairment-focused goal statements should, when possible, be replaced by functional-focused ones, in part because this is in line with the World Health Organization's International Classification on Functioning, Disability and Health (41), but mostly because functional-focused goals are related to the impact of spasticity in each individual patient (42, 43).

It is important to consider the time frame of each goal to ensure it is realistic, attainable and able to demonstrate progression in treatment-induced recovery. An adequate mixture of short- and long-term goals will keep the patient engaged with their individualized management programme and inform the multidisciplinary team whether the treatment approach is working and when it needs to be adjusted. This direction gives a common focus for the rehabilitation team (44). The likelihood of achievement increases when adequate short-term goals are used, even when challenging long-term goals are set, because the existence of short-term goals keeps the patient motivated and compliant with the long-term rehabilitation programme and goals. Therefore, when setting goals, a suggested process involves first negotiating the desired goal based on the patient's current situation, the situation they would like to progress to, and the team's estimation of the realistic probability of that achievement with the available resources and within the time frame of one/several treatment cycles. This is followed by the setting of specific (short- or long-term) goals and the planning of how these individualized goals can be achieved, in addition to ensuring that the patient has coping strategies and the confidence to allow the plan to be implemented. The final stages of the goal setting process involve actioning the agreed plan and then appraising and recording feedback on goals so that adjustments can be made and new goals can be set as appropriate (45).

In the context of BoNT-A treatment, short-term goals should cover only one treatment cycle of BoNT-A (3–6 months). Long-term goals should allow for at least 3–4 BoNT-A treatment cycles (9–18 months).

Based on expert recommendations, shoulder spasticity goals can be grouped into five categories, with some goals applicable to multiple categories (Table 3):

- Passive or symptom-control goals: reducing pain and discomfort, which interfere with sleep and positioning (affecting walking or wheelchair posture). Reducing or even preventing spasms (frequency and severity);
- Passive function goals (tasks performed by others or by the unaffected limb instead of the affected one): reducing difficulty or restoring the ability to be dressed or put on/remove and tolerate orthosis/splints, maintaining passive shoulder abduction to allow, for example, improved personal hygiene or participation in physical and/or occupational therapy, and reducing difficulty in positioning in a bed, wheelchair and/or standing-frame;
- Active function goals (tasks performed with the affected limb): reducing difficulty or restoring the ability to dress, maintaining personal hygiene and feed oneself, as well as participating in domestic/leisure/job-related activities and maintaining the ability to be in a job;
- Personal factors: reducing negative impacts on self-image, self-esteem and social inhibition;
- Global mobility: improving postures that may interfere with the capacity for collaboration during mobility in bed, transfers, seat/stand/seat and balance in gait.

**Table 3:** Goals of BoNT-A treatment in the shoulder for patients with upper limb spasticity.

**Goal**	**Short term^a^**	**Long term^b^**
**Symptomatic**	*Reduce pain*	
	Reduce discomfort	
	Reduce/prevent spasms	
**Passive function**	*Restore ability to dress*	
	*Restore ability to maintain personal hygiene*	
	Allow participation in physical and/or occupational therapy	
**Active function**		*Restore ability to dress*
		*Restore ability to maintain personal hygiene*
		*Restore ability to feed oneself*
		*Restore ability to participate in domestic/leisure/job-related activities*
**Personal factors**	*Reduce reactions associated with involuntary movements, including interference with balance*	*Reduce negative impacts on self-image, self-esteem and social inhibition*

a Period: one treatment cycle of BoNT-A (3–6 months).

b Period: at least three to four cycles of BoNT-A (9–18 months); goals are the same as short-term goals, but should be more ambitious.

The goals considered most important are indicated in italics.

BoNT-A, botulinum neurotoxin type A.

The most relevant short-term goals for patients, as identified by the expert panel, are summarized in Table 3.

Setting Specific, Measurable, Achievable, Realistic, Time-bound (SMART) goals for single or multiple treatment cycles may involve breaking some of the patients' goals into steps/stages for progressive improvement/achievement, and is the job of the multidisciplinary team. This can be likened to climbing a staircase, where short-term goals are the steps and the long-term goal is to reach the top stairway. Goals that are unrealistic within the span of one treatment cycle may be achievable after multiple cycles with cumulative improvement/benefits. This point is important to discuss with both patients and caregivers.

## Summary

Due to their involvement in a large range of movements and essential activities for arm/hand functions such as reaching, the shoulder muscles are an important treatment target for managing UL spasticity with BoNT-A to gain improvement in passive and active function in the post-acute and chronic phase following traumatic brain injury and stroke. The choice of muscles to inject with BoNT-A can be based on analyzing the UL spastic pattern, but ideally by considering which activities – shoulder movements or synergies – are impaired and by targeting the overactive muscles involved in that limitation or hurdle, to achieve the goals of the patient. Local treatment guidelines and product labels must also be considered.

Common patterns of shoulder spasticity seen after stroke will only be treated effectively by targeting all of the inappropriately activated contributing muscles. Correcting this basic coactivation is the first step to reducing pain and increasing range of movement, preserving skin hygiene and integrity, improving posture and facilitating active movement, when present. Multi-pattern BoNT-A injections may modulate sensory feedback to improve comfort and allow progress with other therapeutic modalities, namely those chosen by the multidisciplinary team.

Goal setting should be patient-centric, realistic, achievable and timed, and may include both short- and long-term goals. Both goal-setting and a goal-oriented plan of action should involve the patient, informal carers and the multidisciplinary neurorehabilitation team.

In part two of this practical guide, we address the BoNT-A injection technique and the choice of outcome measurement tools, as well as providing case studies to demonstrate applicability in routine clinical practice.

## Author contributions

All authors were involved with the conception and choice of approach for the expert consensus, contributed findings from their knowledge, and experience to the expert consensus. All authors drafted the manuscript, critically revised the manuscript for important intellectual content and approved the final version of the manuscript.

## Funding

This expert consensus was funded by Merz Therapeutics GmbH.

## Conflict of interest

Authors AC-B and JJ have received honoraria from AbbVie/Allergan, Ipsen and Merz, as scientific advisor, clinical researcher, lecturer, moderator, and peer trainer. Author SC has received honoraria from AbbVie/Allergan and Merz, as scientific advisor, lecturer, and trainer. Author DH has received honoraria from Merz as a scientific advisor. Author JW has received honoraria from AbbVie/Allergan, Ipsen, Merz, Medtronic and Shionogi as scientific advisor, clinical researcher, lecturer, and moderator or peer trainer. The authors declare that this study received funding from Merz Therapeutics GmbH. The funder arranged the expert consensus meetings, reviewed the manuscript and was involved in the decision to submit it for publication.

## Publisher's note

All claims expressed in this article are solely those of the authors and do not necessarily represent those of their affiliated organizations, or those of the publisher, the editors and the reviewers. Any product that may be evaluated in this article, or claim that may be made by its manufacturer, is not guaranteed or endorsed by the publisher.
